# A Survey on Fused Filament Fabrication to Produce Functionally Gradient Materials

**DOI:** 10.3390/ma17153675

**Published:** 2024-07-25

**Authors:** Arup Dey, Monsuru Ramoni, Nita Yodo

**Affiliations:** 1 Department of Computer Science and Engineering Technology, Valdosta State University, Valdosta, GA 31698, USA; 2 Department of Manufacturing and Industrial Engineering, The University of Texas Rio Grande Valley, Edinburg, TX 78539, USA; 3 Department of Industrial and Manufacturing Engineering, North Dakota State University, Fargo, ND 58102, USA; nita.yodo@ndsu.edu

**Keywords:** additive manufacturing, composites, functionally gradient materials (FGM), fused filament fabrication (FFF)

## Abstract

Fused filament fabrication (FFF) is a key extrusion-based additive manufacturing (AM) process for fabricating components from polymers and their composites. Functionally gradient materials (FGMs) exhibit spatially varying properties by modulating chemical compositions, microstructures, and design attributes, offering enhanced performance over homogeneous materials and conventional composites. These materials are pivotal in aerospace, automotive, and medical applications, where the optimization of weight, cost, and functional properties is critical. Conventional FGM manufacturing techniques are hindered by complexity, high costs, and limited precision. AM, particularly FFF, presents a promising alternative for FGM production, though its application is predominantly confined to research settings. This paper conducts an in-depth review of current FFF techniques for FGMs, evaluates the limitations of traditional methods, and discusses the challenges, opportunities, and future research trajectories in this emerging field.

## 1. Introduction

Materials have undeniably been vital in meeting an extensive range of human needs and serving diverse purposes. With engineering and technology advancing rapidly and customer demands constantly evolving in a fiercely competitive market, the necessity for a wide variety of materials with diverse properties has increased. Through practical exploration, scientists have unveiled numerous natural materials and their wide-ranging applications, including various natural functionally gradient materials (FGMs) alongside pure materials and composites [1,2,3]. For instance, FGM bamboo has been used for a long time for decorations and construction [4,5,6]. Additionally, bones and teeth serve as remarkable examples of natural FGMs that perfectly meet distinct functional demands [7,8]. To fulfill the specific demands of various engineering applications like aerospace and automobile industries, creating human-made FGMs has become essential. Human-made FGMs are engineered materials that exhibit a gradual and continuous variation in composition, microstructure, or properties within a single part [2]. FGMs are typically composed of multiple constituent materials, which can include metals, ceramics, polymers, or their combinations, and are designed to have a smooth transition from one property or characteristic to another, typically to optimize performance or functionality in specific applications [3]. Moreover, a single-material FGM can achieve a gradual and continuous variation in properties through alterations in microstructure, density, infill structures, etc. This comprehensive review article concentrates on the fabrication processes and advancements in human-made FGMs to meet the ever-evolving needs of these industries.

Composed of multiple material constituents, FGMs can be considered a type of composite material. Composite materials are made by blending filler materials with matrix materials [9,10,11]. In a composite, the ratio of constituents is equal throughout the cross-section [12,13]. This characteristic allows composite materials to acquire distinct functional properties, including mechanical, thermal, and other attributes that surpass the properties of their constituents. For instance, the properties of a composite with PLA matrix Copper (Cu) particle filler are from properties of both polylactic acid (PLA) and Cu [14,15]. A high strength-to-stiffness ratio, wear resistance, and high reliability are some unique characteristics that can be obtained from composites [16,17]. In composite materials, properties are inhomogeneous throughout the cross-section or sudden changes in properties, not gradually varying [18]. Other limitations are delamination and a sharp change in properties between two different constituents [18]. On the other hand, the properties can be controlled over dimensions with varying microstructure, compositions, and design in FGMs [8,19,20]. In FGMs, cost, properties, weights, and dimensions can be optimized by varying properties to meet specific requirements of an intended function [21,22,23].

The early development of FGMs originated in the 1980s. The “functionally gradient material” concept was first introduced in 1984 in Japan to prepare high-performance heat resistance materials for a space plane project [24]. However, the development progress of FGMs is slow due to the limited fabrication processes [25]. Most traditional manufacturing methods cannot produce FGMs, and the limited number of available techniques to create FGMs makes it challenging to manufacture sizeable components. Creating a material with a graded composition requires precise control over the fabrication process. In addition, the complexity of FGMs, such as the gradient of material properties, also poses challenges in terms of material characterization and testing, which can further slow down the development process.

Functionally graded materials (FGMs) are advanced engineering materials characterized by variations in compositions and structures to obtain novel properties for specific functional requirements [18,19]. FGMs exhibit distinct properties and densities across various regions and orientations. This characteristic enables FGMs to dynamically adapt their material response to deformation and dynamic loading, tailoring it to specific requirements within different sections of a given FGM component. Nowadays, researchers worldwide are increasingly dedicated to advancing the development of FGMs in response to growing demands across diverse fields [19]. FGMs have garnered significant interest and application in energy conversion systems [26]. Notably, as early as 1987, various production methods were introduced in a pioneering space project aimed at FGMs’ development [24]. Over time, several conventional techniques have been established for fabricating FGMs, broadly categorized as gas-based, solid-phase, and liquid-phase methods. Each of these techniques has its unique characteristics and limitations. 

In recent decades, the concept of additive manufacturing (AM) has revolutionized the production of intricately shaped components through additive manners. Just like numerous other industries, additive manufacturing processes have found utility in the creation of FGMs. Several additive manufacturing processes, including fused filament fabrication (FFF), selective laser sintering (SLS), vat polymerization, material jetting, and laser powder bed fusion, are used for FGM fabrication [27]. The use of AM processes for FGMs is still in the early stages, and researchers are exploring the strengths and weaknesses of different AM processes. 

Numerous research endeavors have focused on the utilization of additive manufacturing processes for the development of FGMs in addition to other conventional techniques for developing FGMs [19,27,28,29]. There are several review articles, including Refs. [28,29,30], that focus on the fabrication of FGMs using various additive manufacturing processes, though not specifically on FFF, to summarize the findings of research efforts. Additionally, there are review articles focused on metal additive manufacturing [27] and biomedical applications [31]. Some review articles also focus on specific additive manufacturing processes. For instance, Nohut et al. [32] published an article on using vat photopolymerization for FGMs, while Yan et al. [33] focused on laser metal deposition. To the best of the authors’ knowledge, there is no article on FGMs specifically focused on the FFF process. This review article aims to comprehensively investigate the advancements made in the FFF process to fabricate functionally gradient components. It encompasses an analysis of conventional techniques, highlighting their respective limitations. Special attention will be given to exploring the distinctive capabilities of the FFF method for FGMs, as well as discussing its applications. Lastly, based on the strengths and limitations of the FFF process, this article will offer insights into future research directions in FFF application for FGM development.

**Figure 1:**
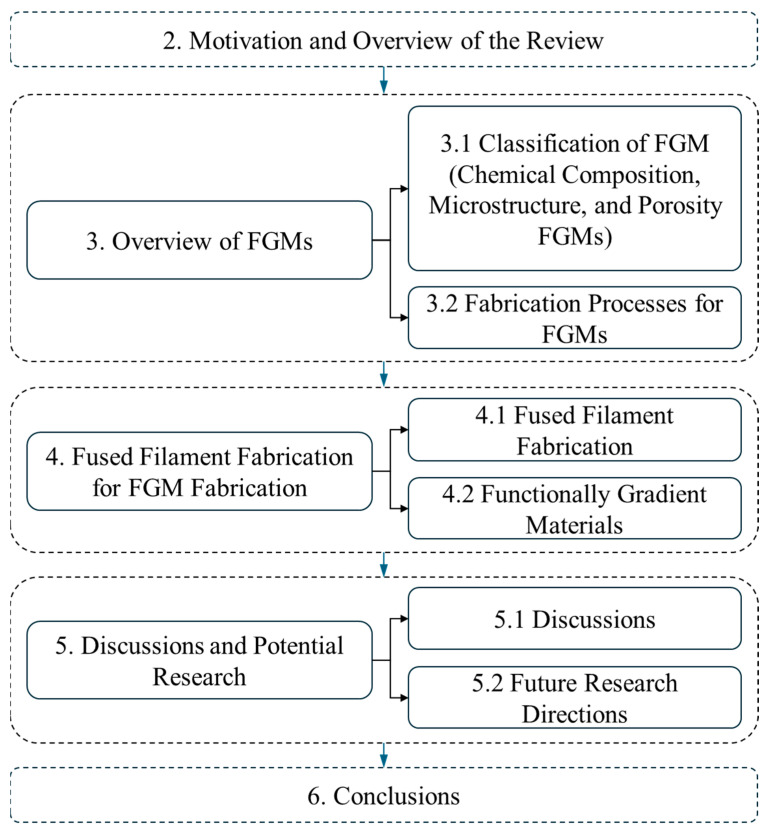
A flowchart of this literature review.

Figure 1 represents the structure of this literature review and a summary of different sections of this literature review. Section 2 presents the motivation and overview of this review article. Section 3 introduces an overview of the types of FGMs and fabrication processes. Existing research on the fabrication of FGMs by the FFF process is rigorously introduced in Section 4. In Section 5, discussions on the literature review and potential research areas for the future are summarized. This literature review article culminates with Section 6, Conclusions.

## 2. The Motivation and Overview of This Review

The motivation to conduct a survey on the applications of the FFF process for functionally graded materials (FGMs) stems from the growing interest in advanced manufacturing technologies that can meet the complex demands of modern engineering and material science. As a versatile and cost-effective additive manufacturing technique, the FFF process offers significant potential for producing FGMs, which are materials with spatially varying properties tailored to specific functional requirements. Despite the expanding research in this area, a comprehensive review is needed to consolidate the diverse findings and highlight the practical applications and future directions of FFF in creating FGMs. This review aims to bridge the knowledge gap by systematically analyzing recent advancements, identifying key challenges, and showcasing innovative solutions that FFF provides for fabricating FGMs. By synthesizing current research trends, industrial applications, and technological innovations, this article will serve as a valuable resource for researchers and practitioners.

Additive manufacturing processes have the capability to produce intricate geometries using a variety of materials, including polymers, metals, composites, and resins, without tooling requirements [34]. Furthermore, they effectively reduce assembly requirements. There have been many efforts to produce functional gradient materials by changing material proportions in different regions, internal density and structures, particle sizes, and many more. Most research efforts focused on developing functionally gradient materials are centered on metal-based additive manufacturing (AM) processes, notably direct energy deposition and powder bed fusion. This emphasis is driven by the considerable demand and the greater availability of research funds from various sources, particularly for aerospace and automobile applications. Fused filament fabrication (FFF) stands out as one of the most extensively utilized additive manufacturing (AM) processes for creating both prototypes and functional components. Topology optimization, process parameter analysis, machine learning applications, and in situ condition monitoring are some research areas related to the FFF process [35,36,37]. Additionally, the exploration of composite materials for the FFF process, achieved through blending metal powder, polymers, biobased fibers, and synthetic fibers with various polymers, represents a prominent area of research interest [9]. Research into the development of functionally gradient components by the FFF process is comparatively limited when compared to efforts in other domains. This review article aims to provide comprehensive insight into the development of FGMs using the FFF process. In pursuit of this objective, this review will commence with a brief introduction to FGMs. Furthermore, it will explore the potential applications of FFF in fabricating functionally gradient parts across various fields. This review will summarize current research endeavors in the development of FGMs through the FFF process, aiming to identify both challenges and opportunities. Additionally, it will explore the existing literature to assess the strategies employed in overcoming these challenges. After conducting an extensive review of existing publications, a series of future research directions will be proposed for researchers. 

In this review article, relevant peer-reviewed publications on the application of fused filament fabrication (FFF) in functionally gradient materials (FGMs) were identified through a comprehensive search on Google Scholar using specific keywords and their combinations. The search keywords included “Fused Filament Fabrication”, “Fused Deposition Modeling”, “Extrusion-Based Additive Manufacturing”, “FFF”, “FDM”, “Functionally Gradient Material”, and “Functionally Gradient Component”, among others. Articles published from 2019 were selected based on an examination of abstracts, journal quality, duplication, and other relevant criteria. This review rigorously discusses various techniques employed to develop FGMs using FFF. It addresses the challenges encountered by researchers and highlights innovative solutions that have been devised. Furthermore, this paper summarizes the limitations of the FFF process, the capabilities and constraints of FFF machines, and the standard testing techniques used in the development of diverse FGM components.

## 3. Functionally Gradient Materials 

**Figure 2:**
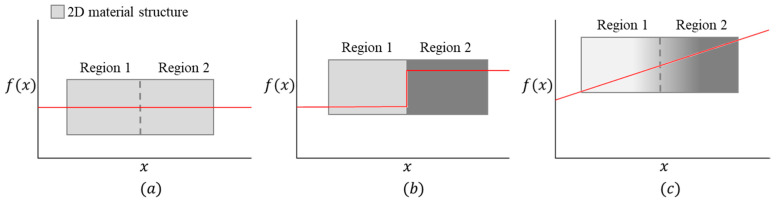
Depiction of structural property behavior: (**a**) uniform, (**b**) sharp change, and (**c**) gradual change.

Pure materials, metal alloys, and composite materials are used in engineering applications to meet different requirements. Pure metals have limited engineering applications that require conflicting properties [38]. For instance, the rigid inner core and hard outer surface are requirements for a gear wheel. Metal alloys, a combination of at least a metal and other metal or non-metal elements, are used instead of pure metal to overcome the limitations. Steel is an iron and carbon alloy with more engineering applications than pure iron metal. To improve the properties of steel, other elements, such as chromium (Cr), vanadium (V), and cobalt (Co), are blended with iron and carbon. Metal alloying is not always convenient for two elements with a large melting temperature gap [39]. Pure metals typically have consistent properties throughout their structure, as shown in Figure 2a. In Figure 2, x represents different structure positions, and f(x) represents a material property (e.g., tensile strength) at x. 

However, diverse engineering applications necessitate varying properties in different areas and orientations. For example, in the case of a cutting tool, it is essential to possess a combination of toughness, hardness, and heat resistance, particularly in the cutting-edge region. Composite materials are advanced engineering materials that improve the properties of their constituents and obtain unique properties [9,40]. In a composite, a matrix and filler are blended at various propositions to obtain tailored properties, improved from the matrix and filler [41,42]. The properties of a composite depend on several factors, including the bond between the matrix and filler, filler direction, filler length, filler proportion, and additives [9,43,44]. The distribution and arrangement of constituents within a composite can result in anisotropic properties, meaning the properties can vary based on the measurement direction. For instance, in a fiber-reinforced composite, the properties are typically stronger and stiffer along the fiber direction while potentially weaker in other directions. This can lead to a distinct or sharp property change at the junction due to lamination, as shown in Figure 2b. Additionally, an abrupt change in properties may occur at the interface between two layers in a laminated composite. Lamination is commonly observed under extreme conditions (e.g., high load) due to weak bonding between the matrix and filler. It is worth noting that properties in composites do not change gradually over their dimensions but rather exhibit localized variations influenced by the composite’s structure and constituent distribution. In composites, properties exhibit uniformity throughout the cross-section given in each direction.

To address the limitations mentioned earlier, FGMs have emerged as promising solutions. FGMs exhibit a gradual variation in material properties, as shown in Figure 2c, which is predetermined to fulfill specific functional requirements. The gradual change in property in FGMs can be achieved by controlling composition, microstructure, and porosity in different directions and positions within the material to meet specific functional requirements [29,45]. Unlike pure materials, alloys, and composites, FGMs do not have uniform properties throughout their volume. Instead, they exhibit a gradual change in properties to perform specific functions in different regions. In FGMs, property variations exhibit discontinuity, where interfaces manifest during shifts in composition, microstructure, and porosity [22].

### 3.1. Classification of FGMs

FGMs can be broadly categorized into three types based on the techniques employed to achieve gradual property change: composition gradient, microstructure gradient, and porosity gradient [31,46]. The subsequent subsections will delve into each of these categories of FGMs.

#### 3.1.1. Chemical Composition FGMs

Chemical composition FGMs represent a category where the properties gradually vary in different spatial positions. These FGMs are created by blending two or more elements, and the gradual property variation is achieved by adjusting the ratio of these elements [47]. This process involves transitioning between different elemental compositions or introducing various materials or phases. The bonding strength between elements and fabrication techniques, such as cooling rate and heat treatment, can influence the occurrence of phase changes within the FGMs. The purpose of composition FGMs is to achieve specific property variations based on the changing composition, such as mechanical strength, thermal conductivity, or chemical reactivity.

**Figure 3:**
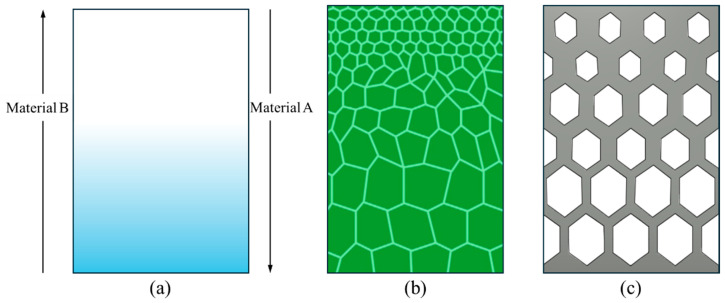
The three categories of FGMs: (**a**) chemical composition, (**b**) microstructure, and (**c**) porosity FGMs.

Compared to pure materials and laminated composites, FGMs achieve smooth transitions in properties by controlling composition and process parameters. In specific applications, material coatings are used for corrosion resistance, but they may suffer from issues such as peeling. FGMs can address this problem by providing a smooth property transition through controlled changes in phases and compositions. A graphical representation of a composition FGM is shown in Figure 3a, where the proportion of Material A decreases from top to bottom, while conversely, the proportion of Material B increases. 

#### 3.1.2. Microstructure FGMs

Microstructure FGMs feature a gradual change in the material’s internal microstructure or crystallographic arrangement [48]. Microstructure gradient is a class of FGMs in which different properties (e.g., hardness and ductility) at different spatial positions are achieved through varying microstructures [49]. This includes variations in grain size, crystal orientation, or the distribution of different phases. Generally, microstructure FGMs are fabricated by controlling heat flow during solidification processes. For example, the quenching heat treatment process can produce hard surfaces due to the martensite microstructure of a part. On the other hand, the inner core will be relatively ductile. A microscopy image of a microstructure FGM is given in Figure 3b. It is visible that the fine microstructure is at the top, and the coarse structure is at the bottom part. The properties are also different in different regions due to microstructure. Another observation is that the transition from one microstructure to another is smooth, not sharp. As a result, the properties will vary gradually, and no stress will be developed in the interfaces. By manipulating the microstructure, microstructure FGMs can achieve desired properties such as improved fatigue resistance, enhanced toughness, or tailored electrical conductivity.

#### 3.1.3. Porosity FGMs

The third category of FGMs is the porosity gradient, which involves a systematic variation in the porosity or void content within the material. The graded porosity is applied to vary the properties in the spatial position, leading to a gradient that affects properties like density, mechanical strength, and thermal conductivity [50]. The properties of the porosity FGM can be varied by controlling the volume of porosity, size, and shape [46]. A porosity FGM is demonstrated in Figure 3c, where the porosity (hexagonal) is decreased from the bottom to the top. Porosity FGMs are often utilized to control properties such as light weight, energy absorption, or thermal insulation by adjusting the distribution of voids or pores within the material.

Porosity FGMs can be classified into two categories based on the methods used to control their properties: density gradient and pore size graduation [46]. In the density gradient, the volume or proportion of porosity is varied to gradually change properties. On the other hand, pore size, particle size, and shape are altered in pore size FGMs. Porosity FGMs are often useful for biomedical applications, for instance, bone implantation [51]. 

It is possible to apply multiple concepts from the composition, microstructure, and porosity variations in the same part to achieve functionally gradient physical properties [52]. In addition, FGMs can be classified into two categories based on the material used:

- Homogeneous FGMs: Homogeneous FGMs are produced using a single material. The gradient in properties is achieved by controlling factors such as microstructure and porosity. The base material can be pure metals, pure polymers, metal alloys, or composites. In homogeneous FGMs, there is no change in the chemical composition; rather, the variation in properties is due to changes in the physical structure of the material. Generally, microstructure and porosity FGMs are considered homogeneous FGMs because their gradients are achieved without varying the proportions of different materials.
- Heterogeneous FGMs: Heterogeneous FGMs involve a gradient in properties achieved through variations in chemical composition. These FGMs are produced using two or more different materials, with the proportion of each material varying at different spatial positions. This results in a gradient of properties tailored to specific application requirements. Chemical composition FGMs are always considered heterogeneous FGMs due to the inherent variation in material composition.

### 3.2. Fabrication Processes for FGMs

FGMs aim to obtain specific properties in a spatial position to meet functional requirements. It is vital to select an appropriate fabrication process to produce FGMs as the fabrication processes significantly influence the quality, properties, and gradation [3]. In numerous instances, specific properties such as corrosion resistance and electrical/heat insulation are required on the surface of a component. Thin-film FGM processing methods are applied to add the necessary properties to the surface of FGMs [53]. On the other hand, in applications with extreme operating conditions, it is often necessary to have graded properties throughout the entire volume of a part, which can pose a challenge in the fabrication of functionally gradient materials. In these applications, processing methods to produce bulk FGMs are used. Each type of FGM employs specific techniques to achieve the desired property gradients. Composition FGMs achieve property changes by varying the ratio of constituent materials, while microstructure FGMs manipulate the internal structure or crystallographic arrangement. Porosity FGMs, on the other hand, control the density, inner structures, particle sizes, and distribution of pore sizes. The fabrication process of FGMs poses significant challenges, and conventional methods are commonly used to produce these advanced materials.

**Figure 4:**
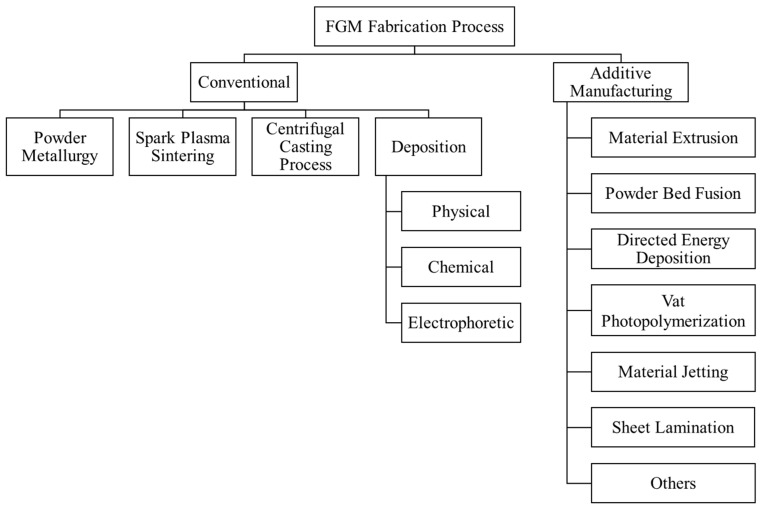
A breakdown summary of various FGM fabrication processes.

Figure 4 provides a summary of both conventional and additive manufacturing processes utilized in the fabrication of FGMs. Many conventional processes are used to produce thin-film and bulk functionally graded materials. Despite the successful application of conventional methods in fabricating FGMs, their scope for applications and quality enhancement remains limited compared to pure materials and traditional composites. Processes such as powder metallurgy (PM), physical vapor deposition (PVD), chemical vapor deposition (CVD), spark plasma sintering (SPS), centrifugal casting (CC), and electrophoretic deposition (EPD) exhibit certain limitations in FGM production. A detailed discussion of various conventional techniques for producing FGMs falls outside the scope of this article. However, the advantages and disadvantages of the conventional processes in developing functionally gradient materials are summarized in Table 1.

Additionally, the limitations of conventional techniques are summarized below.

- Limited process control and scalability. Conventional techniques such as PVD, CVD, and EPD face challenges in accurately controlling the detailed thickness and continuous composition of the coating during deposition. In SPS, the short-range diffusion of atoms may affect the uniform production of FGMs during the sintering process when powders are consolidated into a solid material through rapid heating and the application of pressure. Furthermore, the CC process relies on centrifugal force, which can lead to non-uniform gradient distribution. Additionally, while FGMs can be successfully produced on a laboratory scale, there are significant hurdles in achieving mass production, ensuring process repeatability, maintaining part quality for functional applications, enhancing process capability, and addressing reliability issues for industrial-scale production.
- Limited geometrical complexity and precision. Conventional techniques for FGMs face limitations in producing geometrically complex parts, like traditional machining processes. Furthermore, these techniques often exhibit restricted gradient variations. For example, centrifugal casting (CC) is suitable primarily for cylindrically shaped parts [50]. Achieving high precision in part production is also challenging. For instance, ensuring homogeneous particle distribution and precise outcomes pose difficulties in the CC process for functional applications [19]. Although PM is widely used for FGMs, achieving greater precision through proper diffusion remains a challenge. Additionally, the presence of porosity in parts produced by PM renders their properties insufficient for specific applications.
- Limited material diversity and gradients. Conventional techniques predominantly focus on metals, metal alloys, and ceramics for developing FGMs, with limited exploration of polymers, polymer composites, and biomaterials. Additionally, most conventional techniques are primarily used for fabricating chemical composition and microstructure FGMs, while there is a need for advancements in developing porosity FGMs. Processes such as CC and SPS are not suitable for achieving a porosity gradient structure. Furthermore, in chemical composition FGMs fabricated through powder metallurgy, sharp changes from one phase to another can lead to issues such as delamination and crack formation. These limitations highlight the importance of exploring diverse materials and developing effective techniques to facilitate the development of more advanced FGMs selection.
- Limited application scope and real-world implementation. The use of conventional techniques for fabricating FGMs is often limited to surface coatings and functional parts in industries such as aerospace, automobiles, military, and energy. These methods, such as PVD, CVD, and EPD, are primarily employed for achieving gradient surface properties through varying processing parameters and material proportions. However, there are constraints when it comes to expanding FGM applications to fields like biomedicine, specifically for bone implants and prostheses, where functionally gradient properties are vital for mimicking living organs. Conventional methods focusing on surface gradients and metal/metal alloys may not be suitable for developing FGMs tailored for biomedical applications.
- Limited cost-effectiveness and sustainability. Conventional techniques for fabricating FGMs often involve expensive tooling requirements. For example, the CC process necessitates costly molds tailored to the specific part shape. It is imperative to explore more affordable fabrication methods to promote research and enable the development of FGMs with a broad spectrum of gradient properties for diverse applications. Furthermore, the sustainability aspect is often overlooked in the fabrication of FGMs. Energy efficiency, environmental considerations, and safety issues are not adequately addressed. Gas-based techniques like CVD, for instance, are energy-intensive and generate hazardous gases during FGM production.

**Figure 5:**
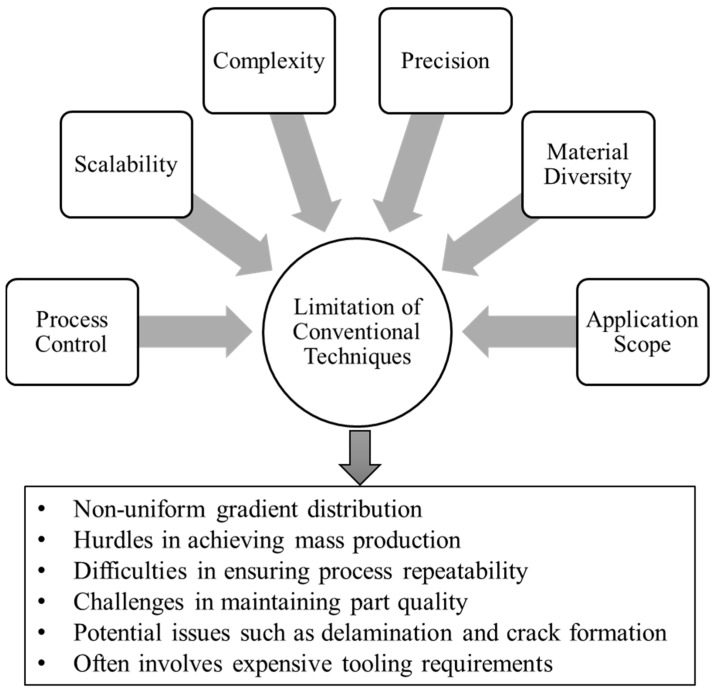
Limitations of conventional techniques for FGM fabrication.

The various limitations associated with conventional techniques, as well as the subsequent consequences that arise from these limitations, are comprehensively summarized and illustrated in Figure 5. Although there are many successful efforts in applying conventional methods for FGMs’ development, there are significant research scopes for improving part properties, increasing material varieties, producing complex parts, and designing different part structures, among other areas. To address this challenge, researchers are exploring new manufacturing techniques, such as additive manufacturing. Due to their unique features, AM processes hold great potential for fabricating FGMs [3,18]. Research on the applications of AM processes for FGM production is in the early stage. Researchers are exploring the viability and advantages of different AM processes for FGM fabrication. Various AM processes, such as material extrusion, which include common techniques known as fused deposition modeling (FDM) and fused filament fabrication (FFF) [54,55], powder bed fusion (PBF) [56,57], directed energy deposition (DED) [58,59], vat polymerization, material jetting [60,61], and sheet lamination (SL) [62], have been applied to analyze the applications for FGMs. Each process presents its own set of advantages and shortcomings. This review is focused exclusively on the FFF process, which is categorized as material extrusion methodology. The subsequent sections provide a summary of the FFF process and its applications in developing FGMs.

## 4. Fused Filament Fabrication for FGM Fabrication

### 4.1. Fused Filament Fabrication

**Figure 6:**
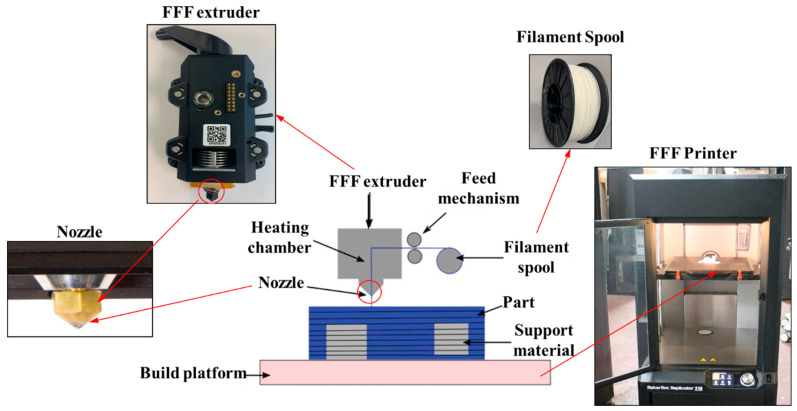
A comprehensive illustration of the fused filament fabrication process.

Fused filament fabrication (FFF) is a popular AM process capable of producing complex parts from polymers and their composites. Among different materials, acrylonitrile butadiene styrene (ABS) and biodegradable polylactic acid (PLA) are the two most popular filament materials for the AM process [9]. A schematic diagram of the FFF process is given in Figure 6. A filament is the most widely used form of material for the FFF process, but pellets can also be used as input materials [63]. The filament is melted in the extruder and deposited through the extruder nozzle according to a predefined toolpath [64]. 

In the FFF process, the deposition of materials can be achieved using different configurations of extruders. In Figure 6, one extruder is used for single-material deposition. Porosity FGMs can be produced using a single extruder FFF machine by adjusting infill density and structures.

However, for multiple-material deposition, either multiple extruders or single extruders with multiple openings are employed. The utilization of multiple extruders or extruders with multiple openings enables the deposition of multiple materials, facilitating the FGMs with chemical composition gradients. These multi-material FFF machines are specifically designed to accommodate the production of FGMs with varying compositions along their cross-sections. 

The FFF process is cost-effective due to the affordability of materials and equipment. It also requires fewer safety precautions compared to metal-based AM processes and those AM processes that involve the use of acids for post-processing. Additionally, FFF (fused filament fabrication) machines, also known as desktop 3D printers, can be set up in various environments, including offices, homes, and classrooms. These machines are relatively compact and often designed for ease of use, making them suitable for a wide range of settings. The FFF process is one of the most used AM processes for the fabrication of FGMs layer by layer. The different research efforts on developing FGMs are discussed in the following section.

### 4.2. Fused Filament Fabrication-Based Functionally Gradient Materials

Different techniques were applied to produce FGMs via the FFF process. These techniques include altering material composition, adjusting process parameters, and modifying infill size, shape, and porosity. Changing material proportions at different spatial positions and adjusting infill parameters and lattice structures are the two most commonly used techniques. Other techniques are also employed. This section is divided into three subsections based on the different techniques used to produce FGMs via the FFF process. 

#### 4.2.1. Material Proportion

One effective approach involves altering the material composition throughout the build, allowing for variations in mechanical, thermal, and chemical properties. By incorporating diverse filament types or blending materials, distinct sections of a part can manifest customized characteristics ideal for their intended purpose. Table 2 presents a comprehensive overview of research endeavors dedicated to the development of FGMs achieved through the manipulation of material proportions utilizing the fused filament fabrication (FFF) process.

Wang et al. [65] produced chemical composition FGMs using 30 μm aluminum nitride (AlN) particles and two sizes of boron nitride (BN) particles (30 μm and 10 μm) as fillers in polycaprolactone (PCL) thermoplastics. The proportions of fillers were varied to achieve gradient variation in thermal conductivity. In the experimental study, three fillers were used separately with PCL for FGM preparations, and the proportion of AlN and BN were varied from 0 to 70% and 0 to 50%, respectively. The maximum number of fillers for FFF materials was determined based on their notched impact strength. In this research, a feed mechanism was designed to guarantee the consistent feeding and mixing of materials by predetermined proportions. In the extruder heating chamber of the FFF machine, variable proportions of constituents were supplied through two openings. It was shown that the particle size, particle shape, and proportion of fillers are vital for achieving gradient thermal conductivity. The temperature in the application area is also important for varying properties. The study revealed that thermal conductivity increased with higher filler proportions and planar particle size. Property measurement is more challenging for gradient parts compared to pure polymers and composites. The same research group analyzed the mechanical properties and porosity of FGMs produced from PCL with AIN and BN fillers [66]. Several pre-processing techniques were implemented to strengthen the bonding between the various constituents. It was shown through experimental investigation that tensile strength increases with increasing the proportions of AlN and BN up to a certain level that decreases after. The porosity increases with the increasing proportions of fillers. The interlayer gap created while printing FGM parts by a multi-material FFF machine is a vital reason for porosity. The filler material’s proportion and the lattice structure of constituents are vital for reducing porosity, which significantly impacts properties. Different additives and surface processing can increase bonding and improve filament properties. The research findings suggest that, depending on specific requirements, it is possible to achieve varied properties across different regions of a component by adjusting filler proportions and materials accordingly. Furthermore, material deposition rates can be regulated by modifying the material feeding mechanism. 

Zhuang et al. [67] used an Isun3d 230C FFF machine to fabricate FGMs, and two filaments at different ratios can blend in the extruder’s heating chamber of the FFF machine. In the research, conductive graphene-doped PLA (G-PLA) was mixed with pure PLA at different ratios at different spatial positions to achieve gradient electrical conductivity. A computer program controlled the deposition rate of PLA and G-PLA. Thermal gravimetric analysis (TGA) revealed that printing temperatures above the melting point of PLA enhance fluidity and decrease viscosity, thereby facilitating improved printing and graphene doping. In addition, strong bonding between PLA and G-PLA was investigated by SEM analysis, and conductivity increases with increasing G-PLA proportions. Therefore, electrical conductivity in pure thermoplastics can be achieved by mixing graphene. Tey et al. [68] integrated a single-screw extruder and an FFF machine to fabricate FGMs. Programmable logic controllers (PLCs) were applied to control the speed of a step motor that controls the rate of material deposition from the FFF machine through the single-screw extruder. The two materials can be blended at various ratios within the hopper of a single-screw extruder, allowing for the production of functionally gradient materials with diverse chemical compositions.

Hasanov et al. [54] used a voxel printing technique to print FGMs by the FFF process from PC and ABS. In our research, Voxolizer Version 2.01 software was employed to vary the proportions of PC and ABS at different locations, aiming to achieve gradient properties. The transition pattern plays a crucial role in determining the quality of a part, with the gradient interface transition pattern taking precedence over other patterns. It was demonstrated through micrographic investigation that the microstructure varies with the changing proportions of PC and ABS. Therefore, achieving a gradient in microstructure is possible by varying the proportions and types of materials. The build orientation influences not only the geometric accuracy of the part but also the distribution of materials within the part, which is crucial for the fabrication of functionally graded materials.

Thermoplastic polyurethane (TPU), a flexible material, was blended with chopped carbon fiber polyethylene terephthalate glycol (CF-PETG), a high-impact strength material, in varying proportions to produce FGMs [69]. Voxilizer software was employed to determine the proportion of TPU and CF-PETG at various spatial positions, and design specimens were subsequently printed using a Zmorph printer for the analysis of tensile and fatigue properties. Experimental analysis revealed decreased tensile properties with increasing TPU proportion, while build orientation significantly affected fatigue. An intriguing observation was that by varying the proportions of both constituents, different properties could be finely tuned.

Li et al. [70] developed a 3D printing mechanism to produce FGMs using the FFF process. For this purpose, they simultaneously implemented a material feeding mechanism and a twin-screw extruder material mixing mechanism instead of relying on filament development. A C++ software package was developed to control feeding and mixing mechanisms. In this experimental study, FGMs were fabricated by mixing conductive materials with PLA to investigate surface insulation performance.

Su et al. [71] blended PLA and ABS at different ratios at different spatial positions to print FGM parts. X-ray computed tomography analysis uncovered a significant disparity in void presence, with the gradient part exhibiting notably higher levels compared to its pure PLA and ABS counterparts. This discrepancy primarily arises from challenges in achieving optimal bonding between successive layers and between the two different materials used in the fabrication process. The gradient properties are achieved only in the Z-direction of printing, wherein the proportion of materials is varied to attain gradient characteristics. Tensile property analysis was conducted as part of this study, underscoring the significance of optimizing process parameters to enhance printing quality.

In addition, Leu et al. [72] developed a method to produce FGMs using a non-conventional FFF process known as Freeze-form Extrusion Fabrication (FEF). In FEF, a printing environment temperature of −20 °C was maintained to prevent deformation from heat stress. The research developed a triple-extruder mechanism with three stainless steel cylinders to produce a chemical composition FGM. To assess the mechanism’s efficacy, FGM parts were printed by mixing alumina (Al_2_O_3_) and zirconia (ZrO_2_), and energy-dispersive spectroscopy (EDS) was used to confirm compositional changes across the graded parts. Li et al. [73] developed FGMs by blending ceramics and refractory metals to investigate their effectiveness in overcoming the high brittleness limitations of ceramics for aerospace applications. For this study, FGM parts were fabricated using the FEF process with zirconium carbide (ZrC) and tungsten (W) for flexural property analysis. A co-sintering post-processing technique was applied to increase the relative density of the printed parts before flexural testing. Experimental investigation showed that a maximum relative density of 81% could be achieved through this post-processing.

Li et al. [74] investigated the effectiveness of Ceramic On-Demand Extrusion (CODE) to produce FGMs. In this experimental study, Al_2_O_3_ and ZrO_2_ paste was blended at different proportions at different spatial positions to produce FGM. Viscous colloidal suspensions of both materials were used as feedstock. Three types of gradient specimens were printed, with alumina proportions increasing from 50% to 100% in increments of 5%, 10%, and 30%, respectively. The printed hardness test samples were sintered in an electric furnace before conducting Vickers hardness tests. EDS analysis showed that the average difference between the actual and desired compositions was about 1%.

#### 4.2.2. Infill Parameters and Lattice Structures

By adjusting infill parameters, including density, size, pattern, and lattice structures, at various spatial positions, a gradient in properties can be attained. Similarly, altering porosity proportions and shapes also facilitates the creation of property gradients. The research endeavors aimed at achieving gradients through these techniques are consolidated in Table 3.

Silva et al. [75] utilized the FFF process to fabricate functionally gradient parts. In this study, internal structures and their dimensions were modified in CAD design to create functionally gradient structures. Honeycomb, lotus, and honeycomb with plateau borders, each featuring distinct structures, were strategically placed at various locations on the samples. This placement involved adjusting the thickness and length of internal structures to achieve gradient properties through two different methods. Subsequently, the designed parts were printed using the FFF process with PLA materials for property analysis and comparison with simulation models. It was shown that the experimental analysis results differ from the simulation results because the printed parts are anisotropic, whereas the simulation was performed with isotropic assumptions.

In an experimental study by Platek et al. [76], four types of gradient parts were designed by changing the sizes and directions of honeycomb structures in different spatial positions to analyze compressive properties through quasi-static uniaxial compression tests. The Zortrax M200 FFF machine was used to print specimens according to the design using thermoplastic co-polymer (TPC) filaments. The investigation suggests that high stiffness can be achieved through a two-directional gradient in the size of the honeycomb cell structure.

Elenskaya and Tashkinov [77] created heterogeneous lattice structures with varying porosity sizes and shapes using different numerical models. Instead of printing parts from the designed structures, finite element analysis (FEA) was applied to analyze properties by using the properties of a PEEK filament as input parameters. It was demonstrated that the stress resulting from abrupt gradient changes and dimensional errors in scaffolds can be reduced by employing proposed numerical design models. A Bievolutionary Structural Optimization (BESO) algorithm was employed by Pais et al. [78] to generate a functionally gradient distribution in a component aimed at maximizing the stiffness-to-weight ratio. In the research, a gyroid infill pattern was optimized, and simulations were conducted to compare with experimental outcomes. For the experimentation, the FFF process was applied to print parts using a PLA filament based on the optimized gradient design. The deviation between experimental and simulation outcomes is 16% when a four-point bending load is applied to a specimen.

Wen et al. [79] designed three types of functionally gradient structures by linearly varying the cell strut diameters of three lattice structures: cubic, honeycomb, and bamboo in the Z-direction. The lattice structures were designed using CAD software, and the quasi-static compression test specimens were printed from PEEK filament by OMNISY H600 FFF machine. Three uniform lattice structures were also printed to compare compressive properties, energy absorption, and deformation behaviors with three FG structures. The study demonstrated that energy absorption is higher for functionally graded structures, while both honeycomb and bamboo structures exhibit superior properties compared to cubic structures in both uniform and FGM structures. The experimental study revealed that layer-by-layer crushing was the predominant failure mode, starting with the thinner strut layer followed by the thicker strut layer sequentially in the FGM lattice structures.

Duraibabu et al. [80] printed FGM parts from ABS plus filament by varying the density and pore size at different positions. In this research, pore shapes were also varied using different cellular structures. An FGM part is divided into five regions by varying density along the Z-direction. Among hexagonal, triangular, square, and cylindrical cellular structures, the hexagonal structure outperforms the others in terms of energy absorption and compressive strength.

#### 4.2.3. Miscellaneous Gradients

Most FGMs are created either by adjusting material proportions at different spatial positions or by altering parameters and lattice structures. Several other techniques are also employed. In this ‘Miscellaneous Gradients’ section, all other techniques used to develop FGMs by the FFF process are discussed. Modifications to process parameters, such as extrusion temperature, print speed, and layer thickness, play a crucial role in defining the properties of the final product. For instance, adjusting the extrusion temperature can influence the adhesion between layers and the overall strength of the part. Furthermore, lamination-type gradients can also be achieved through FFF processes. The research efforts in this area are summarized in Table 4.

Srivastava et al. [81] produced an FGM component by changing process parameters such as raster angle, raster width, contour width, and air gap. The gradient component is produced by changing density in different regions. In the research study, ANSYS 14 software was employed to analyze the effects of transverse load on deformation. The analysis revealed a noteworthy reduction of 51% in deformation. By considering the impact of load in various regions, it is possible to optimize both the load-bearing capacity and the cost of production by selecting different combinations of parameters for each region. 

In a study by Jaiswal et al. [84], a surrogate-based optimization model was developed to estimate a build orientation that minimizes material composition and geometric error. In this research, the staircase effect and the discretization across the cross-section of the toolpath were considered the reasons for geometric errors and material composition errors, respectively.

Caliskan et al. [82] utilized the FFF process to fabricate functionally gradient materials in graded sandwiched layers of two filament materials, ABS and PLA. The proportions of PLA gradually increased from 10% to 90%. It is well known that printing temperature and other process parameters are different for ABS and PLA. This factor was taken into consideration when printing functionally gradient parts. Specifically, the printing temperature and build platform temperature were increased, and the cooling rate was decreased when the proportion of ABS was 20% or higher.

Palaniyappan et al. [55] developed FGMs by using PLA and walnut-reinforced PLA (WPLA) filaments. In this research, both filaments were produced instead of using commercial filaments. A total of 10 layers of specimens were printed. CATIA V5R20 and CURA were used for CAD design and slicing, respectively, in their study. In one type of FGM, five consecutive PLA layers and five consecutive walnut-reinforced PLA (WPLA) layers were printed. In the second type of FGM, three PLA layers, four WPLA layers, and three PLA layers were printed. Finally, in the third type of FGM, three WPLA layers, four PLA layers, and three WPLA layers were printed. The printed samples resemble those prepared by the sheet lamination process with two materials. Different mechanical, thermal, and tribological properties of the FGM samples were analyzed and compared with pure PLA and pure WPLA samples. Experimental investigation revealed that the tensile, flexural, and compressive properties of the first type of FGM surpass those of pure WPLA. While some properties may degrade compared to PLA and WPLA, FGM samples exhibit unique combinations of different properties that are crucial for various functional applications.

Ritter et al. [83] developed an additive manufacturing device capable of producing functionally graded high-temperature thermoplastic PEEK materials by manipulating their microstructure during manufacturing. Five strategies for controlling chamber temperature and crystallinity were explored and compared, including hot air delivery, dual-nozzle design, ambient temperature control and enclosure, heated plate, and supplementary infrared heaters. Through the experimental printing of tensile test specimens, it was demonstrated that high-quality parts with gradient properties can be successfully produced.

Garland and Fadel [85] conducted extensive investigations to understand the limitations of current FFF machines, STL files, G-code, and slicers for printing functionally graded materials. To achieve uniform properties throughout a printed part, ensuring the consistent mixing of materials is crucial within the heating chamber of an extruder equipped with one nozzle and two openings. 

FFF can overcome several limitations in fabricating FGMs, as discussed in Section 3. The main benefit of FFF in producing FGMs is the ability to vary the composition and properties of the printed object easily. By using different types of filaments with varying properties, FFF can create FGMs with graded mechanical, thermal, and electrical properties, among others. Another known FFF is the ability to create complex geometries with intricate shapes and geometries as well as with high accuracy and precision that conventional processes may not achieve easily. FFF is a relatively simple and low-cost AM technology compared to other methods, making it accessible for a broader range of FGM applications.

## 5. Discussion and Potential Research

### 5.1. Discussion

FGMs find applications in diverse fields such as aerospace, medicine, defense, manufacturing, energy, and optoelectronics [39,86,87,88,89,90,91]. The specific properties required for a material vary depending on the intended application. As previously discussed, extensive research has been conducted to fabricate FGMs using different techniques, including various AM methods. 

It is important to highlight that AM methods are not intended to replace conventional methods but serve as complementary approaches in fabricating FGMs. When considering a specific application, it becomes crucial to conduct thorough experimental analyses to evaluate the advantages and disadvantages of AM compared to conventional techniques or hybrid approaches combining AM and conventional methods. These analyses aim to identify the critical factors contributing to enhancing part properties and successfully fabricating FGMs using each method. This review aims to make a significant contribution to the field of FGM fabrication by thoroughly exploring the intricacies of the FFF process. The insights gained from this review are intended to advance the development of FGMs and support the ongoing research and innovation in additive manufacturing technologies. By understanding the strengths and limitations of the FFF process, researchers can make informed decisions and leverage the synergies between these approaches to optimize the fabrication process and advance the development of FGMs.

### 5.2. Future Research Directions

**Figure 7:**
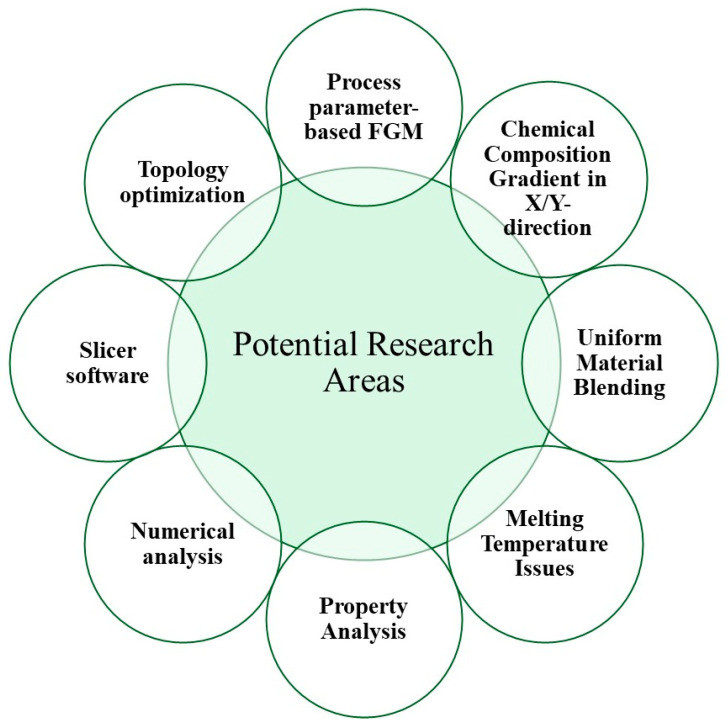
Future research directions for developing FGMs by the FFF process.

While the research efforts into producing FGM components via the FFF process were limited, the exploration undertaken by the research community yielded numerous intriguing findings and promising directions. Based on an intensive literature review, the findings are summarized in the previous sections, and future research directions are introduced below. Additionally, Figure 7 highlights potential future research directions for advancing the development of FGMs using the FFF process.

**Figure 8:**
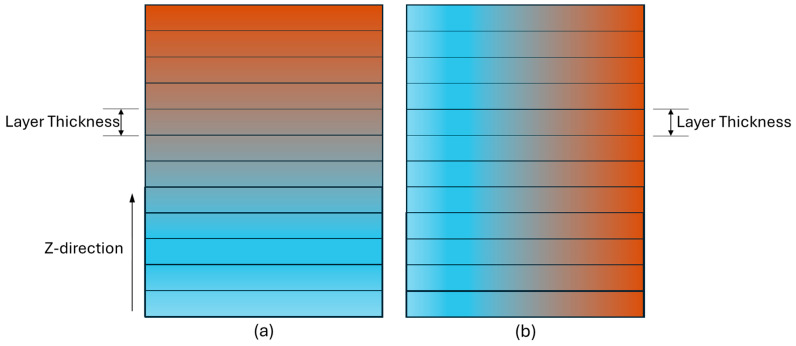
Gradient (**a**) along Z-direction and (**b**) along other directions.

1. Process parameter-based FGM: The FFF process parameters (e.g., layer thickness, infill density, build orientation, infill structures, print speed, etc.) have significant impacts on part qualities, build time, and costs. Based on requirements, different combinations of process parameters can be used at different spatial positions to optimize properties, build time, and costs. For this, optimization techniques such as multiobjective optimization and topology optimization can be used. Customized G-code is required to control print speed, deposition rate, and other parameters at different spatial position. Many open-source software options are available to customize G-code for printing parts via the FFF process.
2. Chemical composition gradient in X/Y-direction: In the FFF process, the chemical composition gradient can be achieved only in the Z-direction as the ratio of two or more materials is constant in a layer. Figure 8a shows the chemical composition gradient along the Z-direction of an FFF machine, while the gradient along another direction is shown in Figure 8b. Different properties (e.g., tensile properties) are low in the Z-direction compared to the X- and Y-directions due to a weak bond between two consecutive layers. Intensive research is necessary to achieve chemical composition gradients in directions other than the Z-direction. One concept involves the continuous mixing of materials at different ratios within the heating chamber of the FFF machine extruder rather than relying solely on filaments. This approach necessitates a customized FFF machine. Another concept involves the simultaneous production of filaments by connecting a twin- or single-screw extruder with an FFF machine. Some research endeavors have focused on customizing FFF machines to be compatible with an extruder for FGM components. Achieving this requires precise control and accuracy in determining the volume of materials at different locations within a layer and adjusting ratios continuously throughout the layer.
3. Uniform material blending: An extruder with one nozzle for material deposition and multiple openings for input filaments is used in the FFF process where material is mixed in the heating chamber of the extruder. In many FFF machines, achieving uniform material mixing can be challenging. As a result, the actual deposited material ratio may differ from the targeted ratio, significantly impacting the quality of an FGM component. To address this challenge, focusing on refining the design of FFF machines is a highly effective approach.
4. Melting temperature issues: When two materials melt within a single chamber, variations in melting point temperatures lead to differences in their molten states, viscosity, and flowability. These property variations will significantly impact factors such as dimensional accuracy and bonding strength. It is vital to consider this factor in material selection for FGM production. When the melting point temperature difference is high for two materials, an FFF machine with multiple extruders can be used. An alternative option may be a preheating system design for materials with high melting points based on the physical, chemical, and other properties of filament materials.
5. Property analysis: Testing the properties of FGM parts is also challenging due to the lack of sufficient standards and gradient features. In destructive testing, determining the physical properties of a specific spatial position is not feasible. While simulation analysis is practical, most simulation software is not designed for additive manufacturing environments, where additional complexity arises due to layer-by-layer processes and the impacts of process parameters. An important research direction involves advancing computational models and simulation techniques to effectively predict and optimize the properties of FGMs produced through AM [81,92]. This entails developing improved design methodologies that leverage the distinctive features of AM processes to achieve tailored material gradients, enhanced performance, and functional integration within FGM structures.
6. Numerical analysis: Most of the research efforts on the development of FGMs are experimental to study the viability of a technique for the successful fabrication of FGMs. Along with the experimental analysis, different optimization techniques [93] and machine learning algorithms can be used to predict properties and set a combination of parameters to obtain the desired properties [94]. For this, a standard data collection step is required to collect robust and high-quality data. Different types of sensors (e.g., vibration sensors, accelerometers, and thermistors) and the design of experiments can be used to collect data.
7. Slicer software: Slicer software presents various limitations. A more effective solution involves modifying an existing slicer to interpret not only the geometric model but also the internal composition model, thus generating a G-code that integrates both. While such an enhanced slicer is currently unavailable, a workaround has been developed to partially address the issue.
8. Topology optimization: Topology optimization optimizes material layout within a given space to achieve the maximum performance of a structure or component. Topology optimization can be utilized to generate functionally graded components by optimizing material distribution. Additionally, it allows for the incorporation of other modifications such as infill structures, adjusting infill density, varying layer thickness, and so on.

FFF processes hold immense potential to fabricate high-quality FGMs for real-world applications in diverse fields. However, establishing FFF as a reliable approach for FGM fabrication still requires further research and exploration. The suggested future works aim to address FFF’s existing challenges and limitations in FGM production, such as the need for more efficient and cost-effective processing techniques and the development of advanced material systems [95]. By pursuing these research directions, FGM production can continue to advance and provide solutions to real-world applications.

## 6. Conclusions

AM processes are viable options to produce FGMs with improved quality and a wide variety for diverse applications. AM processes are relatively inexpensive and straightforward without expensive tooling requirements for many applications, particularly in biomedical fields. This review article discusses the potential and limitations of the FFF process in fabricating FGM components. Initially, it introduces the three types of functionally gradient materials that can be produced using the FFF process. Subsequently, the limitations of various conventional methods are summarized to highlight the objective of employing the FFF process for FGM materials. The ongoing research on developing FGMs by the FFF process is rigorously reviewed to assess progress and pinpoint research gaps in the field. The research gaps for successfully developing functionally gradient materials are identified based on the current research efforts and their pros and cons. These findings can guide interested researchers and practitioners in identifying future research directions and opportunities for applications of FFF for FGMs. In the future, different AM processes, specifically FFF, will be applied to fabricate FGMs. The impacts of AM process parameters will also be analyzed using data analysis techniques based on experimentally collected data.

## Figures and Tables

**Table 1:** Advantages and disadvantages of conventional processes in fabricating FGMs.

Methods	Advantages	Disadvantages
Powder metallurgy (PM)	Ability to control particle size and distribution Capability to produce metal-based complex shapes	Limited control over compositional gradients Difficulties in achieving continuous gradients Potential for interfacial reactions or contamination
Centrifugal casting (CC)	Effective for producing FGMs from liquid-phase matrix materials Continuous compositional gradients Good control over material distribution	Limited to producing tubular-shaped FGMs Challenges in achieving other complex shapes Potential for segregation and compositional variations
Spark plasma sintering (SPS)	Rapid densification from powder particles Flexibility in controlling temperature and pressure Potential for cost-effective mass production	Limited control over compositional gradients Challenges in achieving complex geometries Difficulty in achieving precise control over porosity distribution
Physical vapor deposition (PVD)	High purity of deposited material Good adhesion to the substrate Control over film composition and coating thickness	Limited scalability for large-scale components Challenges in achieving graded compositions Expensive equipment and complex process Limited only for surface gradients
Chemical vapor deposition (CVD)	Control over deposition rate and film thickness/composition Good adhesion and high coating density Ability to achieve desired dimensional accuracy	Complex and costly equipment Limited scalability for large-scale production Difficulties in achieving continuous compositional gradients Limited only for surface gradients
Electrophoretic deposition (EPD)	Ability to fabricate graded compositions Good control over particle deposition Potential for complex shapes and customized properties	Limited scalability for large-scale components Potential for defects and porosity in deposited layers Limited only for surface gradients

**Table 2:** Current research on gradient via material proportion.

Ref. (Year)	Gradient Techniques	Material	FFF Machine	Technology/Equipment	Analyzed Properties
Wang et al. (2020) [65]	Material proportion	PCL, BN, AIN	-	-	Impact strength, thermal conductivity
Zhang et al. (2021) [66]	Material proportion	PCL, BN, AIN	m-FDM	HAAKE twin-screw (Thermo Disher Scientific Inc., Karlsruhe, Germany) Extruder, DMA Q800 (TA Instruments, New Castle, DE, USA), universal testing machine	Interlaminar shear strength, storage modulus, and tensile strength
Zhuang et al. (2017) [67]	Material proportion	PLA and G-PLA	iSUN3d 230C (ESUN3D Printing Co., Ltd., Shenzhen, China)	QUANTA 450 (FEI, Hillsboro, OR, USA), TGAQ600 (TA Instruments, New Castle, DE, USA), DM3068 (Rigol Technologies, Suzhou, China), APS3003S-3D (Shenzhen Atten Technology Co., Ltd., Shenzhen, China)	Electrical conductivity and heat distribution
Tey et al. (2019) [68]	Material proportion	PA66	-	PLC, single screw extruder	Controllable mixer design
Hasanov et al. (2020) [54]	Material proportions at voxel scale	ABS and PC	ZMorph Fab Multi-Tool (Zmorph S.A., Wrocław, Poland)	Voxolizer, Instron 5582 UTM (Instron, Noorwood, MA, USA), FEA simulation	Microstructure, tensile strength, Young’s modulus
Alkunte et al. (2022) [69]	Material proportions at voxel scale	TPU and CF-PETG	ZMorph Fab Multi-Tool	Voxolizer (Version 2.01)	Tensile and fatigue properties
Li et al. (2020) [70]	Material proportion	$\mathrm{PLA},\;Al_{2}O_{3},TiO_{2}$ $SrTiO_{2}$	--	C++ (feeding and mixing mechanism), twin-screw extruder), dielectric spectroscopy analyzer	Surface insulation performance
Su et al. (2021) [71]	Material proportion	PLA and ABS	GEEETECH A30M (HK Getech, Co., Ltd., Shenzhen, China)	X-ray (tomography)	Young’s modulus and natural frequency

**Table 3:** Current research on gradient via infill parameters and lattice structures.

Ref. (Year)	Gradient Techniques	Material	FFF Machine	Technology/Equipment	Analyzed Properties
Silva et al. (2022) [75]	Infill structure’s size and shape	PLA (experiment) PLA and Aluminum–PLA (simulation)	UltiMaker 3 (UltiMaker, Zaltbommel, The Netherlands)	SolidWorks 2018, CURA (Slicer), NX Nastran V2019.1 (simulation), Instron 3369 (Instron, Noorwood, MA, USA)	Flexural properties
Platek et al. (2019) [76]	Infill structure’s size and shape	TPC	Zortrax M200 (Zortrax, Olsztyn, Poland)	MTS Citerion C45 (MTS Systems GmbH, Berlin, Germany)	Stress and deformation energy
Elenskaya and Tashkinov (2021) [77]	Porosity size and shape of lattice structure using geometric models	PEEK (simulation)	--	Abaqus (FEA)	Elastic behavior using FEA
Pais et al. (2021) [78]	Gyroid infill pattern optimization by BESO	PLA	Creality ender 3 (Shenzhen Creality 3D Technology Co. Ltd., Shenzhen, China)	FEMAs software (gyroid law)	Flexural properties
Wen et al. (2021) [79]	Strut diameters of lattice structures	PEEK	OmniSys H600 (Omni Tech Suzhou Co. Ltd., Suzhou, China)	LE 3504 UTM (LiShi Co., Shanghai, China), Abaqus	Compressive properties, energy absorption, and deformation behaviors
Duraibabu et al. (2020) [80]	Size and shape of infill structure	ABS plus	U-print SE (Stratasys, Ltd., Edina, MN, USA)	Universal testing machine	Energy absorption and compression strength

**Table 4:** Current research on miscellaneous gradient techniques.

Ref. (Year)	Gradient Techniques	Material	FFF Machine	Technology/Equipment	Analyzed Properties
Srivastava et al. (2016) [81]	Process parameters	ABS (simulation)	--	ANSYS 14	Deformation under transverse load
Caliskan et al. (2024) [82]	Lamination sandwich structure	ABS and PLA	GEEETECH A30M (HK Getech, Co., Ltd., Shenzhen, China)	CURA V4.10.0	Tensile properties
Palaniyappan et al. (2023) [55]	Lamination sandwich structure	PLA and WPLA	Pratham 3.0	CATIA V5R20 (CAD), CURA, Universal Testing Machine	Tensile, compression, and flexural properties, contact angle, heat deflection, specific wear rate, coefficient of friction, and fracture morphology
Ritter et al. [83]	Microstructure via thermal control	PEEK	Velleman K8200 (Vellman, Ghent, Belgium)	Hitachi S-4700 SEM machine (Hitachi High-Tech, Tokyo, Japan), CURA V4.3, Arduino, TMP37 temperature sensor (Analog Devices Inc., Middlesex County, MA, USA)	Tensile properties

## Data Availability

Data will be provided upon request to the corresponding authors.
